# Predictors of Unsafe Induced Abortion among Women in Ghana

**DOI:** 10.1155/2019/9253650

**Published:** 2019-02-03

**Authors:** Michael Boah, Stephen Bordotsiah, Saadogrmeh Kuurdong

**Affiliations:** ^1^Ghana Health Services, Bolgatanga Upper East Region, Ghana; ^2^Chinese Centre for Endemic Diseases Control and Prevention, Harbin Medical University, Harbin, Heilongjiang Province, China; ^3^Department of Global Health and Development, College of Medicine, Graduate School of Hanyang University, Seoul, Republic of Korea; ^4^St Patrick Nursing/Midwifery Training College, Offinso, Ashanti Region, Ghana

## Abstract

**Background:**

Unsafe induced abortion is a major contributor to maternal morbidity and mortality in Ghana.

**Objective:**

This study aimed to explore the predictors of unsafe induced abortion among women in Ghana.

**Methods:**

The study used data from the 2017 Ghana Maternal Health Survey. The association between women's sociodemographic, obstetric characteristics, and unsafe induced abortion was explored using logistic regression. The analysis involved a weighted sample of 1880 women aged 15-49 years who induced abortion in the period 2012-2017. Analysis was carried out using STATA/IC version 15.0. Statistical significance was set at p <0.05.

**Results:**

Of the 1880 women, 64.1% (CI: 60.97-67.05) had an unsafe induced abortion. At the univariate level, older women (35-49 years) (odds ratio=0.50, 95% CI: 0.28-0.89) and married women (odds ratio=0.61, 95% CI:0.44-0.85) were less likely to have an unsafe induced abortion while women who did not pay for abortion service (odds ratio=4.44, 95% CI: 2.24-8.80), who had no correct knowledge of the fertile period (odds ratio =1.47, 95% CI: 1.10-1.95), who did not know the legal status of abortion in Ghana (odds ratio =2.50, 95% CI: 1.68-3.72) and who had no media exposure (odds ratio =1.34, 95% CI: 1.04-1.73) had increased odds for an unsafe induced abortion. At the multivariable level, woman's age, payment for abortion services, and knowledge of the legal status of abortion in Ghana were predictors of unsafe induced abortion.

**Conclusion:**

Induced abortion is a universal practice among women. However, unsafe abortion rate in Ghana is high and remains an issue of public health concern. We recommend that contraceptives and safe abortion services should be made available and easily accessible to women who need these services to reduce unwanted pregnancies and unsafe abortion rates, respectively, in the context of women's health. Also, awareness has to be intensified on abortion legislation in Ghana to reduce the stigma associated with abortion care seeking.

## 1. Introduction

Developing countries account for the greatest proportion of the global maternal deaths that occur annually [1]. The maternal mortality ratio in Ghana is 310 per 100000 live births and direct maternal causes account for 67% of maternal deaths [2].

Abortion contributes 15-30% of the maternal mortality in Ghana [3]. The reasons why women induce abortion are well documented [4–8]. Nevertheless, scientific breakthrough has made it possible for women to obtain safe abortion services. Regrettably, unsafe induced abortions prevail. It is estimated that nearly half of the 56 million abortions that occur every year are unsafe and 97% of these unsafe abortions take place in developing countries [9]. According to the World Health Organization (WHO), abortion is unsafe when an unwanted pregnancy is terminated by either a person without the prerequisite skills or a procedure being undertaken in an environment that does not satisfy the minimum medical standards or both [10]. Complications from unsafe abortion account for the largest proportion of hospital admissions to gynaecological wards in developing countries [11]. The criminal code in Ghana was amended in 1985 legalizing induced abortion under certain circumstances [5]. Nevertheless, access to safe abortion services by Ghanaian women is hampered by limited access to legal abortion services, cost, sociocultural barriers, and social stigma [12].

The government of Ghana has attempted to address unwanted pregnancies and unsafe abortions through promoting the use of modern contraceptives, inclusion of abortion services in the Ghana reproductive health strategic plan, and capacity building of trainee midwives in the health training institutions on comprehensive abortion care [3, 13]. In spite of this, contraceptives uptake remains low at 25%, 31% of pregnancies are mistimed or unwanted, induced abortion rate has increased to 7% in 2017 from 5% in 2007, and unsafe abortion is a significant contributor to maternal morbidity and mortality [2, 3, 14].

Many published studies in Ghana have explored induced abortion. However these studies are not nationally representative; thus findings cannot be applied to all women in Ghana [5, 15–17]. Moreover, the literatures on unsafe induced abortion are scarce. Sundaram, Juarez, Bankole, and Singh reported that maternal age, parity, and wealth are associated with obtaining safe abortion services in Ghana using nationally representative data from the 2007 Ghana Maternal Health Survey (GMHS) [18]. In their study, abortion was classified as safe if the woman used a safe method and provider if even the location was not safe. However, morbidity and mortality from unsafe abortion are linked to the method used and the type of provider in addition to the safety of the instruments and environment where the service is undertaken [3]. Therefore, the study by Sundaram and his colleagues is liable to underreporting of the outcome which has implications for planning purposes and decision making.

This study addresses this gap and provides current information on unsafe abortion rate including the profile of women who procure unsafe abortion services in Ghana. The findings will be of policy importance to the Ministry of Health (MoH) of Ghana and other professionals worldwide working in the area of female sexual and reproductive health.

## 2. Materials and Methods

This study used data from the 2017 GMHS. The 2017 GMHS was implemented by the Ghana Statistical Service (GSS) with technical support from ICF through the Demographic and Health Survey (DHS) program. The sampling frame used was from the 2010 Population and Housing Census (PHC) conducted in Ghana. All women aged 15-49 years who were permanent residents of selected households or visitors who stayed in selected households the night before the survey were eligible for interview. A multistage stratified cluster sampling method was used to select enumeration areas and households. The details of the survey procedures and the questionnaires used can be found in the final report [2].

This study has restricted the analysis to a subpopulation of women who have ended a pregnancy between 2012 and 2017 due to the outcome of interest. The household recode, individual recode, and births recode datasets were merged to provide more information on the women including events related to their most recent induced abortion. A total of 1425 women were included in the study. However, in the GMHS survey, the sample was selected with unequal probability and hence reduced sample variability for subgroups for which statistics are required. As a result, adjustment factors such as weights are applied to produce values that are representative [19]. Therefore, our analysis is on a weighted sample of 1880 women aged 15-49 years.

### 2.1. Variables

The dependent variable was safety of induced abortion. It was constructed in a binary form as “0” for “safe” and “1” for “unsafe” from three variables (type of method, type of provider, and location). Firstly, the use of vacuum aspiration, misoprostol, combination of misoprostol and mifepristone, dilation and curettage, or dilatation and evacuation were classified as medical methods. Nonmedical methods included drinking milk/coffee/alcohol/other liquid with sugar, drinking a herbal concoction, drinking another home remedy, using a herbal enema, inserting a substance into the vagina, heavy massage, excessive physical activity, and tablets (exact kind unknown). Secondly, a doctor or a nurse/midwife was classified as a medical provider and the rest were nonmedical providers. Finally, a public government hospital, public government health centre/clinic, private hospital/clinic, private family planning/Planned Parenthood Association of Ghana (PPAG) clinic, and private maternity were considered as safe locations. Therefore, in this study, an induced abortion is considered safe if the pregnancy was terminated by a “medical provider” using a “medical method” in a “medically safe location.” The outcome of interests in this study was unsafe induced abortion defined as the termination of pregnancy by a woman through the use of nonmedical method, nonmedical provider, in an environment that is not medically safe for that purpose.

The independent variables included were age, age at first sexual intercourse, highest educational attainment, respondent's religious affiliation, ecological zone (of residence), place of residence (rural or urban), media exposure, previously induced abortion (whether the respondent had a previous history of induced abortion), multiple steps to end pregnancy (whether respondents made multiple attempts to end the pregnancy), payment for abortion service, knowledge of the fertile period, and knowledge of the legal status of abortion in Ghana and contraceptive use at time of pregnancy. The variable ecological zone was categorized from the 10 regions in Ghana into Northern zone (Northern, Upper East and Upper West Regions), Middle zone (Eastern, Ashanti and Brong Ahafo Regions), and Coastal zone (Western, Central, and Greater Accra Regions). Media exposure was dichotomized: “Yes” for respondents who reported either reading newspapers, listened to radio, watched television or used the internet within the one week reference period in the GMHS, or “No” if otherwise. We categorized women who said the fertile period was “halfway between two periods” as ‘Yes” for correct knowledge about the fertile period and “No” if otherwise. Variables were recoded where necessary to produce a meaningful sample for analysis.

### 2.2. Statistical Analysis

The DHS program uses a complex survey design in its surveys [19]. Hence, individual sampling weights were used to account for the design used in the GMHS. The “svy” command prefix was used in the estimation of means, proportions and confidence intervals (CI). The association between the independent variables and the dependent variable was explored in a univariate and multivariable logistic regression analysis. In the univariate analysis, variables with p values of ≤0.1 were simultaneously included in a multivariable logistic regression model. Statistical significance was set at a p-value of < 0.05. The odds ratio (OR) and adjusted odds ratio (AOR) with their 95% confidence intervals were calculated. All analysis was done in STATA/IC 15.0 for Windows (StataCorp LLC, College Station, Texas USA). The fit of our final model was checked using the “svylogitgof” command [20]. The model fit results showed that there was no evidence of a lack of fit of our model in significantly predicting unsafe induced abortion.

### 2.3. Ethics

The ICF Institutional Review Board (IRB) approved the protocol for the 2017 GMHS. However, ethical approval was not needed for this study since it involved a secondary analysis of a dataset without personal identifiers to respondents and their households. Nonetheless, permission was obtained from ICF for the use of the datasets in this study and the terms of use have been observed.

## 3. Results and Discussion

### 3.1. Results

#### 3.1.1. Background Characteristics of Respondents Who Induced Abortion Recently

The mean age of the participants was 27.64±6.66 years (range 15-48 years). The majority had their first sexual encounter before 18 years (59.9%), were Christians (90.7%), and lived in the urban setting (65.4%). Of the total respondents, 546 (29.0%) attained secondary education or above and 464 (24.7%) were from households belonging to the highest wealth quintile (Table 1).

Regarding abortion behaviour (Table 2), the majority of participants had no previous history of induced abortion (67.5%) and made a single attempt in terminating the recent abortion (86.4%). Of the total abortions, 64.1% (CI: 60.97-67.05) were unsafely induced while 18.7% of the participants reported using contraceptive at the time of pregnancy. The main reason mentioned by many of the women (15.2%) for inducing abortion was ‘No money to care for baby.'

### 3.2. Predictors of Unsafe Induced Abortion

A univariate and multivariable logistic regressions were used to model the predictors of unsafe induced abortion (Table 3). At the univariate level, relatively, women who are 35-49 years old (OR=0.50, 95% CI: 0.28-0.89) and married women (OR=0.61, 95% CI: 0.44-0.85) were less likely to have an unsafe induced abortion whereas women who did not pay for abortion services (AOR=4.44, 95% CI: 2.24-8.80), who did not have correct knowledge of the fertile period (OR=1.47, 95% CI: 1.10-1.95), did not know the legal status of abortion in Ghana (OR=2.50, 95% CI: 1.68-3.72), and who had no media exposure (OR=1.34, 95% CI: 1.04-1.73) were more likely to have unsafe abortion. At the multivariable level, woman's age, payment for abortion service, and knowledge of the legal status of abortion in Ghana remained significant predictors of abortion safety.

## 4. Discussion

This study was designed to explore the predictors of unsafe induced abortion among women in Ghana. The findings show that 64.1% of the induced abortions were unsafe. The proportion of unsafe abortions reported in this study is higher than that reported from earlier nationwide studies in Ghana [18] and Nepal [21]. The differences in proportion of unsafe abortions are attributed to the inclusion of location safety in defining abortion safety in this study.

The findings showed that older women (35-49 years) relative to younger women were less likely to have unsafe abortion which corroborates previous findings from Ghana [18] and other countries [21, 22] contrary to findings from Ethiopia [23]. Younger women are more liable to sexual coercion and rape that can lead to unplanned pregnancies [24, 25] and lack access to contraceptives to prevent unwanted pregnancies [26, 27] and financial resources for childcare [25, 27]. A significant proportion of induced abortions results from unintended pregnancies [28, 29]. Additionally, safe abortion services are not easily affordable in Ghana due to limited legal facilities and practitioners to provide these services [30]. Therefore, when the decision to abort is reached the absence of financial, social, and psychological support drives younger women to opt for cheaper and easier accessible unsafe abortion services. It is reported that women seek safe abortion services when supported financially [18]. Women who did not pay for abortion services in this study had unsafe abortions and this in part rests on the clandestine method used. Cost is also implicated in unsafe abortions in instances when safe abortion services are well-known to women [21, 31–33].

Awareness of the law on abortion can motivate women with unwanted pregnancies to access safe abortion services [34]. In this study, women who did not know the legal status of abortion in Ghana were more likely to procure unsafe abortion services. We attribute this to lack of self-confidence coupled with antiabortion sentiments in the Ghanaian society. In Nepal, after legislation on abortion, women who obtained unsafe abortion services were unaware of the legal status of abortion [35]. Notwithstanding, this finding is contradicted by a recent study in the same country [21]. In that study, however, induced abortion was more common among women who were aware of the legal status of abortion suggestive that other factors such as wealth and social connections might have contributed to the decision on the provider for abortion services [36].

It is worth noting that 18.7% of women in this study who induced abortion were on contraceptives. Though contraceptive failure was not mentioned as a reason for abortion in this study and does not directly facilitate unsafe abortion per se, it underscores its contribution to unwanted pregnancies that result in induced abortion. Misconceptions on the use of modern contraceptives need to be addressed appropriately through sexual and reproductive health education to minimize contraceptive failure especially due to improper use. Also, the debate on the ethics of abortion may continue but without doubt, access to contraceptives and safe abortion services improves health and reduces mortality [34].

Finally, Ghana's abortion law is relatively less restrictive. However, awareness among women and some cadre of health professionals is low [37]. The proportion of women in this study who said abortion in Ghana is legal was 11.4%, an increase from 4% in 2007 [38]. This means that women are becoming knowledgeable about abortion legislation in Ghana though efforts are still required to increase public awareness.

There are some limitations to this study that have to be acknowledged. Firstly, in constructing the dependent variable, we included a measure for location safety. However, misoprostol is a widely available and less expensive medical abortifacient that can be used in any location with or without the assistance of a qualified medical professional. Thus, we classified women who used this method as having used an unsafe abortion service if the procedure was conducted by either a doctor or a nurse/midwife but not in a medically safe location. Nevertheless, a surgical abortion method will require a medically safe environment to prevent infections [39]. Secondly, not all the factors that have a known association with unsafe induced abortion have been explored in this study. Finally, the study used data from a cross-sectional study that involved a recall of events over a 5-year period. This predisposes the information collected to recall bias in addition to underreporting of abortion-related events due to the stigma governing abortion practices in Ghana. The findings should, therefore, be interpreted with caution when drawing conclusions on causality. Notwithstanding, information was elicited from the subpopulation on the most recent induced abortion to minimize recall biases; robust statistical methods were used to identify the predictors of unsafe induced abortion rendering the results reliable and generalizable to women in Ghana.

## 5. Conclusion

We sought to explore the predictors of unsafe induced abortion among Ghanaian women. Woman's age, payment for abortion service, and knowledge of the legal status of abortion in Ghana were significant predictors for unsafe abortion services. It is recommended that modern contraceptives and safe abortion services should be made available and easily accessible to women who need these services in the context of health. Also, public awareness should be intensified on Ghana's abortion law to destigmatize abortion care seeking.

## Figures and Tables

**Table 1:** Background characteristics of respondents who induced abortion recently (N=1880 unless stated).

**Variable/category**	**Frequency**	**Percentage**
**Demographic characteristics**		
*Age (years)*		
<20	145	7.7
20-24	541	28.8
25-34	875	46.5
35-49	319	17.0
*Age at first sexual intercourse*		
<18 years	1126	59.9
18 years and over	754	40.1
*Marital status*		
Single	761	40.5
Married	438	23.3
Cohabiting	681	36.2
*Religious affiliation*		
Traditional	48	2.6
Christian	1705	90.7
Islam	127	6.8
**Socioeconomic characteristics**		
*Highest educational attainment*		
No education	162	8.6
Primary	298	15.9
Junior high	874	46.5
Secondary or above	546	29.0
*Ecological zone*		
Northern	53	2.8
Middle	795	42.3
Coastal	1032	54.9
*Place of residence*		
Urban	1230	65.4
Rural	650	34.6
*Wealth index*		
Lowest	101	5.4
Second	312	16.6
Middle	455	24.2
Fourth	548	29.1
Highest	464	24.7

**Table 2:** Distribution of respondents by abortion characteristics, knowledge/information, and contraceptives use.

**Variable/category**	**Frequency**	**Percentage**
**Abortion characteristics**		
*Previously induced abortion *		
Yes	611	32.5
No	1269	67.5
*Multiple steps to end pregnancy*		
Yes	256	13.6
No	1624	86.4
*Used a medical method*		
Yes	1312	69.8
No	568	30.2
*Used a medical provider *		
Yes	799	42.5
No	1081	57.5
*Used a safe location *		
Yes	754	40.1
No	1126	59.9
*Safety of induced abortion*		
Safe	675	35.9
Unsafe	1205	64.1
*Payment for abortion services *		
Yes	1752	93.2
No	128	6.8
*Main reason for ending pregnancy∗*		
No money to take care of baby	285	15.2
Not ready to be a mother	254	13.5
Wanted to space child	216	11.5
Wanted to continue schooling	195	10.4
Partner did not want child/denied paternity	161	8.6
*Antibiotics taken after abortion*		
Yes	1231	65.5
No	649	34.5
**Knowledge/information **		
*Knowledge of fertile period (1738)*		
Yes	883	50.8
No	855	49.2
*Knowledge of legal status of abortion in Ghana*		
Yes	200	10.6
No	1680	89.4
*Media exposure*		
Yes	1110	59.0
No	770	41.0
**Contraceptive use**		
*Using contraceptive at time of pregnancy*		
Yes	352	18.7
No	1528	81.3

*∗*Only the 5 topmost reasons have been presented.

**Table 3:** Univariate and multivariable logistic regression of the predictors of unsafe induced abortion among women (15-49 years of age) in Ghana.

**Variable/category**	**Univariate**		**Multivariable**	
	**OR**[**95%CI**]	**P-value**	**AOR**[**95%CI**]	**P-value**
**Demographic characteristics**				
*Age (years)*				
<20	1.00		1.00	
20-24	1.13[0.64-2.01]	0.669	1.04[0.55-1.98]	0.897
25-34	0.65[0.38-1.11]	0.112	0.61[0.33-1.45]	0.126
35-49	0.50[0.28-0.89]	0.018	0.47[0.24-0.91]	0.026
*Age at first sexual intercourse*				
<18 years	1.00			
18 years and over	0.93[0.73-1.18]	0.537	-	
*Marital status*				
Single	1.00		1.00	
Married	0.61[0.44-0.85]	0.003	0.78[0.53-1.16]	0.225
Cohabiting	0.90[0.66-1.21]	0.481	0.84[0.60-1.17]	0.304
*Religious affiliation*				
Traditional	1.00			
Christian	0.81[0.31-2.06]	0.650		
Islam	0.74[0.26-2.12]	0.579	-	
**Socioeconomic characteristics**				
*Highest educational attainment*				
No education	1.00		1.00	
Primary	1.32[0.75-2.33]	0.340	1.07[0.58-2.00]	0.817
Junior high	0.96[0.61-1.51]	0.857	0.86[0.49-1.50]	0.584
Secondary or above	0.69[0.44-1.09]	0.108	0.72[0.37-1.40]	0.329
*Ecological zone*				
Northern	1.00			
Middle	1.34[0.91-1.99]	0.143		
Coastal	1.10[0.74-1.63]	0.632	-	
*Place of residence*				
Urban	1.00			
Rural	1.13[0.86-1.48]	0.397	-	
*Wealth index*				
Lowest	1.00		1.00	
Second	1.85[0.93-3.68]	0.077	1.72[0.80-3.74]	0.167
Middle	1.56[0.86-2.83]	0.142	1.49[0.74-3.01]	0.267
Fourth	1.05[0.58-1.87]	0.880	1.19[0.59-2.38]	0.626
Highest	0.74[0.41-1.30]	0.296	1.03[0.51-2.07]	0.943
**Abortion characteristics**				
*Previously induced abortion*				
Yes	1.00			
No	1.29[0.99-1.67]	0.058	1.10[0.83-1.47]	0.498
*Multiple steps to end pregnancy*				
Yes	1.00			
No	1.29[0.90-1.84]	0.164	-	
*Payment for abortion services*				
Yes	1.00		1.00	
No	4.44[2.24-8.80]	<0.001	4.64[2.19-9.83]	<0.001
**Knowledge/information**				
*Correct knowledge of fertile period*				
Yes	1.00		1.00	
No	1.47[1.10-1.95]	0.009	1.29[0.96-1.73]	0.088
*Knowledge of legal status of abortion in Ghana*				
Yes	1.00		1.00	
No	2.50[1.68-3.72]	<0.001	2.06[1.30-3.28]	0.002
*Media exposure*				
Yes	1.00		1.00	
No	1.34[1.04-1.73]	0.023	1.05[0.75-1.49]	0.764
**Contraceptives use**				
*Using contraceptive at time of pregnancy*				
Yes	1.00			
No	1.19[0.86-1.63]	0.291	-	

Goodness of fit test.

F-adjusted test statistic = F(9, 562)=0.268 and p=0.983.

## Data Availability

The data supporting the conclusions drawn in this study have been included in this paper. However, the datasets underlying the findings of our study are publicly available online (www.dhsprogram.com).
